# *Taenia saginata*: A Rare Cause of Gall Bladder Perforation

**DOI:** 10.1155/2012/572484

**Published:** 2012-06-26

**Authors:** Suhail Yaqoob Hakeem, Arshad Rashid, Suhail Khuroo, Rajandeep Singh Bali

**Affiliations:** ^1^Department of Surgery, Government Medical College, Srinagar 190010, India; ^2^Department of Surgery, Lok Nayak Hospital, Maulana Azad Medical College, New Delhi 110002, India

## Abstract

We report a case of biliary peritonitis caused by gall bladder perforation due to *Taenia saginata* induced gangrenous cholecystitis. Although parasites are not unusual causes of biliary tract disorders, especially in disease endemic areas, but this is for the first time that *Taenia saginata* has been reported to cause gall bladder perforation.

## 1. Introduction

Taeniasis is endemic in Southeast Asia. Two species from the genus *Taenia* are common parasites of humans—the pork tape worm or *T. solium* and the beef tape worm or *T. saginata*. Recent studies suggest that the taenia found in Asia is a subspecies of *T. saginata* and it has been renamed as *T. saginata asiatica* [1]. Infection is acquired by taking improperly cooked beef or pork. Most cases of taeniasis are asymptomatic and usually complain of passage of proglottids with stools. However, others present with pruritus ani (77%), nausea (46%), abdominal pain (43%), dizziness (42%), increased appetite (30%), and other mild gastrointestinal symptoms [2]. We report a case of a 32-year-old man with biliary peritonitis caused by gall bladder perforation due to *Taenia saginata* induced gangrenous cholecystitis—a very rare but potentially fatal complication of taeniasis.

## 2. Case Presentation

**Figure 1:**
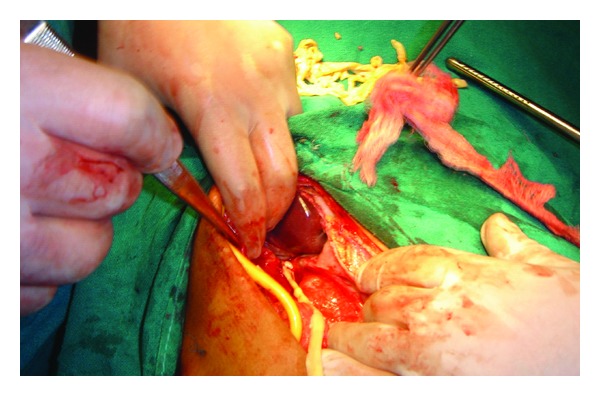
*Taenia saginata* coming out of the gall bladder. Also seen is Foley's catheter used for cholecystostomy.

A 32-year-old unmarried male, smoker, and beef eater from rural Kashmir, India presented to the surgical emergency department of a tertiary care hospital in Srinagar with features of acute abdomen. He had a three-day history of upper abdominal pain of increasing severity, nausea, and bilious vomiting with fever and chills. Previous history was suggestive of chronic acid peptic disorder. Physical examination revealed anicterus, tachycardia, temperature of 38.5°C, tenderness, and guarding in the epigastrium and right hypochondrium. The total leukocyte count was 13.5 × 10^9^/L with a differential count depicting polymorphs_77_, lymphocytes_20_, monocytes_02_, and eosinophils_01_. The total bilirubin was 12.7 *μ*mol/L, ALP was 197 IU/L, AST was 68 IU/L and ALT was 42 IU/L. Chest and abdominal radiographs (standing/supine) were normal. USG abdomen reported free fluid in Morrison's pouch. The patient was operated with an impression of a perforated duodenal ulcer. Operative findings showed a severely inflamed gall bladder with patchy necrosis and a perforation in fundus with pericholecystic pus collection. Further exploration was interesting, and to our surprise we extricated an adult tape worm of approximately 1.7 m in length from the gall bladder which was devoid of stones (Figure 1).

Peritoneal mopping, closure of perforation, and cholecystostomy were done. Cholecystectomy was avoided in view of severe inflammation around the Calot's triangle. Rest of the viscera were normal. The specimen sent to the department of pathology/parasitology was confirmed to be *Taenia saginata*. In the postoperative period, patient had mild respiratory tract infection. Bile started draining with pus flakes from cholecystostomy tube on 5th post operative day. Cholangiogram on 9th postoperative day showed a normal anatomy and free drainage into the duodenum. Liquid orals were started on 2nd postoperative day with full orals resuming by 4th postoperative day. The cholecystostomy tube was removed on 14th postoperative day. Retrospective history was negative for jaundice or passage of worm segments. The patient was given a single dose of praziquantel: 15 mg/kg body weight. Parasitological controls (two series of three fecal samples each), performed two months later, were negative for Taenia eggs. The patient was followed up regularly, and an interval cholecystectomy was done after 6 weeks.

## 3. Discussion

*Taenia saginata* infestation has got a global distribution and is endemic in this part of the world. In a study by Wani et al. [3] conducted in the rural areas of Kashmir, the prevalence of this helminth was reported to be 7.69%. This is possibly due to consumption of undercooked beef as a peculiar dietary habit. Beef contains the larval form of this helminth known as cysticercus. After activation in the upper gastrointestinal tract, the cysticercus attaches to the wall of the small intestine by means of scolices and becomes a mature tapeworm. This maturation process takes 10–12 weeks for *T. saginata* [4]. Owing to this attachment, Taenia would not be expected to migrate in the gastrointestinal tract. However, there have been occasional case reports of finding this helmnith in the main pancreatic duct causing acute pancreatitis [5] and nasal expulsion of this worm along with a nasogastric tube [4] and few reports of finding this worm in the biliary tract and gall bladder causing acalculous cholecystitis [6–11]. To the best of our knowledge, this is the first case reported in the world literature wherein gall bladder perforation had developed due to *Taenia saginata.*

Acute cholecystitis and cholangitis has been associated with a wide range of infectious agents including various helminths. Parasites known to be associated with this condition include *Ascaris lumbricoides *and* Clonorchis sinensis* [12]. These are both wandering helminthes with no attachments in the intestine and can easily traverse the ampulla and reach the biliary tree. Since Benedict [6] reported the first biliary migration of *Taenia saginata*, it has been a matter of speculation as to how Taenia reaches the biliary tree. The presence of Taenia within the biliary channels should be termed as biliary taeniasis. Benedict [6] and Logan [13] believed that the adult form migrates through the ampulla. We presume that after activation of the cysticercus, it may have migrated proximally into the biliary channels before being attached to the small gut, and instead of developing in the small gut, it may have matured in the gall bladder and biliary tree only. These views have been previously expressed by Talice and Perez-Moreira [7]. It has not escaped our attention as to how this helminth can survive and mature in the hostile environment of bile.

**Figure 2:**
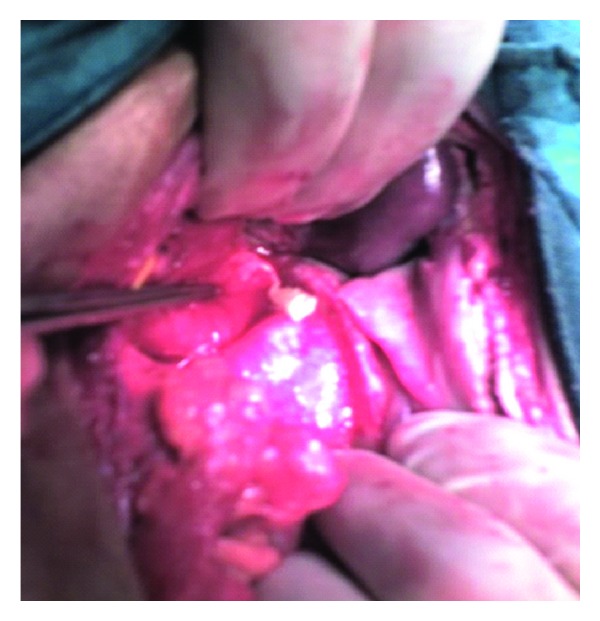
Proglottid of *Taenia saginata* coming out of gall bladder.

Ultrasonography is a simple and noninvasive method for detecting helminthes in the biliary tract and pancreas [14]. ERCP has also been used in the diagnosis and extrication of biliary helminthes [15]. In our case, ultrasonography revealed presence of free fluid in the Morrison's pouch with poorly visualized gall bladder and biliary channels owing to the presence of bowel gases. We operated the patient with the clinical impression of peptic ulcer perforation. But to our surprise, we noticed gangrenous cholecystitis with gall bladder perforation and a proglottid of Taenia coming out of it (Figure 2).

After removal of the worm, we closed the perforation in the gall bladder and did a formal cholecystostomy, as there were dense adhesions at the Calot's triangle. Interval cholecystectomy was done after 6 weeks. We believe that this approach is the most fruitful one as it avoids any inadvertent injury to the inflamed biliary tree.
